# Between proteins and phenotypes: annotation and interpretation of mutations

**DOI:** 10.1186/1471-2105-10-S8-I1

**Published:** 2009-08-27

**Authors:** Christopher JO Baker, Dietrich Rebholz-Schuhmann

**Affiliations:** 1 University of New Brunswick, Saint John, PO Box 5050, Canada, E2L 4L5; 2 European Bioinformatics Institute, Wellcome Trust Genome Campus, Hinxton CB10 1SD, UK

## 

Understanding the roles of genes and proteins and the functional consequences of their mutation is mandatory for the interpretation of a resulting phenotype, for example the observed disease state. Biomedical scientists are approaching this task from different angles. Typically individual research projects investigate the consequences of natural and experimental mutations in proteins by analysing stability and the resulting changes to a protein's function [1]. The consequences of such changes may be used in simulations and subsequent population studies may analyse cohorts for genetic variability, after which review of the observed phenotypes in each of the cohort's individuals may occur [2,3]. Results are reported in the scientific literature and in biomedical databases but are not necessarily integrated according to a detailed and comprehensive knowledge representation, nor are they accessible for reuse by multiple stakeholders.

Annotation of mutations with their relevance for phenotypic expression is crucial to the understanding of genetic mechanisms, biological processes and complex diseases. Large-scale resources able to cope with the full extent of data and annotations reporting on human variability (see 1000 genomes project) have yet to be built [4]. Systems maintaining this data will have to deal with a number of problems: (i) extraction, storage and reuse of genotype-phenotype information [5,6], (ii) preparation of semantic resources for phenotype description (phenotype ontologies, e.g. human phenotype ontology) [7,23], and (iii) automated interpretation, simulation and prediction of functional changes induced by mutations and sequence variants [8].

In principle, a complete solution that allows efficient hypothesis generation based on annotated mutation information would be able to derive, from the location of the mutation, changes in protein structure and function thus allowing prediction of changes in the protein's activity and the molecular processes that are affected. Finally, it would realize and describe the overall changes in the microscopic, physiological and macroscopic phenotype. Such a solution is not yet in sight.

The integration of data from different experimental and simulation methods as well as the annotation of data with rich semantic resources (bio-ontologies [9]) are the most important next steps [10]. To date, moving from SNP to sequence to structure and function has been addressed with varying degrees of accuracy with sequence and structure based methods. The need to apply prediction techniques at a genomic scale requires that adequate solutions have to be identified and benchmarked against reliable measures. Only then can we anticipate and design solutions to address changes expected from significant mutations. In this context the reuse of existing mutations and annotations, from databases and those mined from the literature, for checking the quality of predictions is pivotal.

In order to assess the state of the art in the annotation, interpretation, management of mutations several initiatives and events have recently taken place [11,12]

These events have brought to light the challenges, the existing solutions and relevant expertise within the research community. This supplement to *BMC Bioinformatics *on mutation related IT solutions gives further insight on the current state of research in this important domain. The presented research is mainly concerned with the impact of mutations on protein stability and protein function but also includes reports on attempts to predict the relevance of a protein's modifications in the context of the disease under investigation. Several publications report on the annotation of mutations in the corresponding gene/protein with information extracted from the scientific literature. A number of the studies used specific protein families to demonstrate the usefulness of the findings.

It is clearly an ambitious goal to discover novel knowledge from the literature by contrasting the retrieved content against reference data resources [13]. For the protein kinases, [14] processed a large set of documents to identify mutations and validate the results against against KinMutBase, revealing an overlap of only 52%. In other approaches, contextual information in neighbouring text was used to find functional annotations of protein residues that could be evaluated against UniProtKb [15] or to efficiently retrieve documents reporting on mutations that destabilise G protein-coupled receptors [16]. In the latter case, the reported destabilisation effects were evaluated against sequence based predictors. Contextual information in Medline abstracts was also exploited, using different machine learning based classifiers, to predict the stability and the disease relevance of mutations in lipase and amylase enzymes [17].

Two further publications describe the integration of extracted mutations into solutions that support interpretation of SNPs in a larger context. Mutations can be visualized in a 3D representation of the protein's structure to better understand the induced effects of the mutation through its three-dimensional localisation [18]. In another approach, the impact of changes to a protein's activity due to its mutation can be traced through protein network simulation [2]. The authors use the example of MEK1 activation of Akt to demonstrate the usefulness of this approach. Both publications show the complexity of the integration tasks, reveal new methodologies – based in part on existing infrastructure, and illustrate new biological insights in the context of existing biological models.

In parallel, other researchers seek to predict the consequence of sequence variation on biological processes. One research team has focused on mutations in mitochondrial sequences and their relevance for diseases. They propose and evaluate a scoring function that has been tested against other prediction methods (SIFT, PolyPhen, PHD-SNP, PLHOST) [19]. In another study, changes in a protein sequence and the protein's stability were assessed in the context of changes in the protein's function – which is assumed to be crucial to the development of a disease state [1]. These researchers showed that changes to the protein's stability have an effect on the protein's function, but there are also cases where the function is not altered at all. In a similar approach, another team used features from protein annotations (e.g., from UniProt) to classify mutations so as to predict changes in the protein's function and its relevance for diseases [20]. Clearly, taken in isolation the criterion of large scale change in a protein's stability is not sufficient to distinguish deleterious mutations and neutral variation. Finally, [21] analyse the adaptations of the HIV virus during the drug treatment of the patients. The genetic variability in the virus' protease is evaluated under the evolutionary pressure induced by the treatment of the patients with a varying number of anti-viral drugs.

Altogether, this special issue gives an overview on the scope of ongoing research to exploit resources reporting on genetic variability. The full integration of all such resources is work in progress and clearly a necessary research direction with significant impact on many areas of biomedical science. Through a renewed community focus on life science infrastructure [9,22] there are opportunities to define a roadmap for technology development in this domain and to evolve existing approaches into a robust framework for phenotype prediction, based on annotations and interpretation of mutation data. Clearly many stakeholders and skills sets are required and a strong community focus must be present.

## Competing interests

The authors declare that they have no competing interests.
